# Cold-Active β-Galactosidases: Insight into Cold Adaption Mechanisms and Biotechnological Exploitation

**DOI:** 10.3390/md19010043

**Published:** 2021-01-19

**Authors:** Marco Mangiagalli, Marina Lotti

**Affiliations:** Department of Biotechnology and Biosciences, University of Milano-Bicocca, 20126 Milano, Italy; marina.lotti@unimib.it

**Keywords:** psychrophilic enzymes, GH1, GH2, GH35, GH42, lactose hydrolysis, galacto-oligosaccharides

## Abstract

β-galactosidases (EC 3.2.1.23) catalyze the hydrolysis of β-galactosidic bonds in oligosaccharides and, under certain conditions, transfer a sugar moiety from a glycosyl donor to an acceptor. Cold-active β-galactosidases are identified in microorganisms endemic to permanently low-temperature environments. While mesophilic β-galactosidases are broadly studied and employed for biotechnological purposes, the cold-active enzymes are still scarcely explored, although they may prove very useful in biotechnological processes at low temperature. This review covers several issues related to cold-active β-galactosidases, including their classification, structure and molecular mechanisms of cold adaptation. Moreover, their applications are discussed, focusing on the production of lactose-free dairy products as well as on the valorization of cheese whey and the synthesis of glycosyl building blocks for the food, cosmetic and pharmaceutical industries.

## 1. Introduction

**Figure 1:**
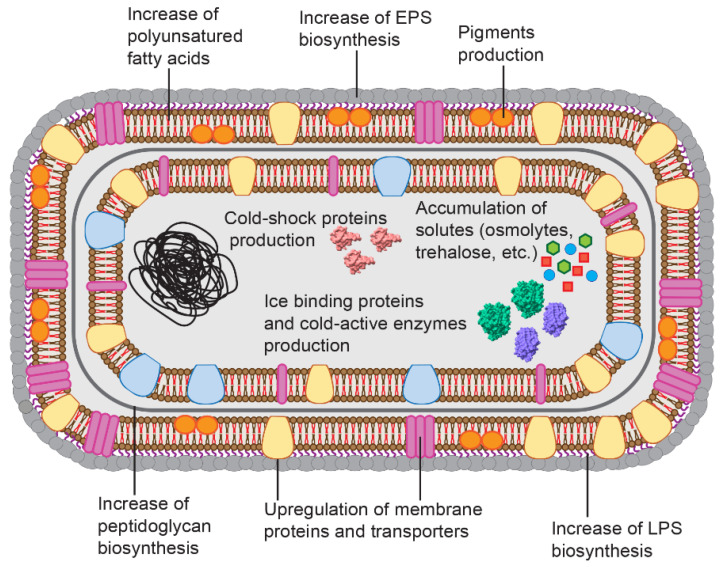
Most common strategies of cold adaptation in bacteria. To survive at low temperatures, bacteria developed several strategies, including the production of cold-shock proteins, ice-binding proteins, cold-active enzymes and compatible solutes. Adaptive changes observed in the inner and outer membranes include the production of pigments (e.g., carotenoids), the upregulation of membrane proteins and transporters and the increase of the biosynthesis of polyunsaturated fatty acids, peptidoglycan, extracellular polymeric substance (EPS) and lipopolysaccharides (LPS).

Cold environments represent a large part of the Earth biosphere [1,2,3]. In particular, polar marine environments, which include seawaters, marine sediments and sea ice, are being studied in depth as a source of bioactive molecules [4]. Microorganisms populating these habitats, mainly bacteria, archaea, protists, unicellular algae and fungi, evolved several physiological and molecular strategies to counteract the multiple stresses to which they are subjected [1,2,3,5]. Among these (summarized in Figure 1), the most common are a peculiar composition of the cell envelope to counteract the decrease of membrane fluidity induced by low temperatures [1,2,3], the production of compatible solutes (e.g., osmolytes) [1,2,3] and ice-binding proteins to prevent the formation of ice crystals and freezing damage [6,7], and of cold-active enzymes required to contrast the negative effect of low temperatures on the rates of metabolic reactions [8,9,10].

Cold-active enzymes are characterized by high activity at low temperatures and are often more thermolabile compared to their mesophilic and thermophilic counterparts [8,9,10]. These two properties make them suitable in processes with heat-labile substrates or products and in those requiring enzyme inactivation, by moderate heating, at the end of the process. Furthermore, the ability of cold-active enzymes to catalyze reactions at low temperature can help to reduce the environmental impact and energy consumption of the process. Cold-active enzymes find application in detergency, waste bioremediation, molecular biology and in the medical, pharmaceutical and food industries [3,4,11,12,13].

**Figure 2:**
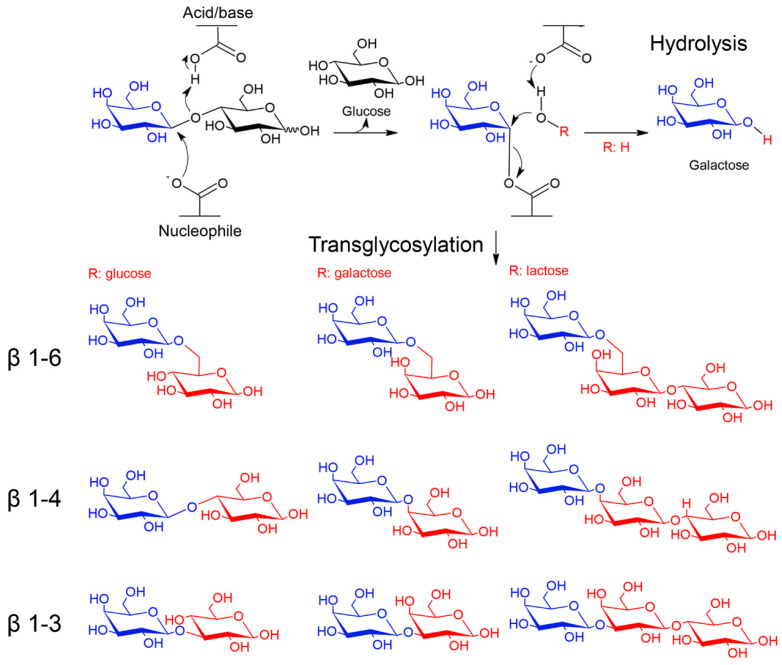
Catalytic mechanism of β-galactosidases. β-galactosidases of families GH1, GH2, GH35 and GH42 use a retaining mechanism of catalysis, which leads to the formation of glycosyl-enzyme intermediate. In hydrolysis reactions the acceptor (ROH) is a water molecule, whereas in transglycosylation reactions is a sugar (e.g., glucose, galactose and lactose) or an alcohol [18]. Examples of transglycosylation products with β-1-6, β-1-4 and β-1-3 galactosidic bonds are shown. The galactose moiety is colored in blue and acceptors in red.

β-galactosidases (EC 3.2.1.23) are glycoside hydrolases (GH) that hydrolyze β-glycosidic bonds of β-galactosides to give galactose molecules. In addition, some β-galactosidases catalyze the transfer of sugar moieties from a sugar donor to an acceptor [14,15]. Because of these activities, β-galactosidases hold great potential in industrial and biotechnological applications [4,12]. These enzymes are widespread and have been isolated from several organisms, including animals, plants, fungi, yeasts, bacteria and Archaea from different environments including the extreme ones [14,15]. According to the CAZy database, which classifies GHs based on the similarities of amino acid sequences, β-galactosidases are grouped in four families GH1, GH2, GH35 and GH42. All these enzymes belong to Clan-A and share a catalytic domain with a (α/β)_8_ TIM–barrel fold and a retaining mechanism of catalysis (Figure 2) [14,15]. Moreover, families GH59, GH147 and GH165 also contain enzymes with β-galactosidase activity.

In this review, we consider β-galactosidases from organisms living in marine cold environments, which enclose an enormous, yet still poorly explored, genetic diversity. Unfortunately, the majority of marine microorganism are unculturable [16,17], which is reflected in the paucity of structural and functional data on marine cold-active β-galactosidases. To shed light on the molecular mechanisms of cold adaptation and to pinpoint the biotechnological exploitation of cold-active β-galactosidases we also discuss enzymes from non-marine environments (i.e., from soils, glaciers and lakes).

## 2. Mechanisms of Cold Adaptation

Cold-active enzymes are suited to counteract the detrimental effect of low temperatures on the rate of chemical reactions. This is an important survival strategy in cold environments, since it has been estimated that a decrease of 10 °C causes a 2–3-fold reduction of the reaction rate [19]. Several studies pointed out that cold-active enzymes can decrease the reaction activation energies compared to mesophilic and thermophilic homologs, which translates into an increase in their catalytic rate (*k_cat_*) at low temperatures at the expense of their *K_M_*. This behaviour relies on reduced enthalpy (i.e., few interactions between enzyme and substrate) and increased entropy (i.e., changes in enzyme stability and flexibility) [8,9,10,19,20,21]. However, in some cold-active enzymes the improvement in the activity at low temperature is obtained through optimization of the *K_M_* [21,22].

All this is obtained by sequence and structural changes that increase the protein structural flexibility. These adaptations may include a peculiar amino acidic composition (i.e., lower content of Pro and Arg residues, and a higher number of Gly residues), a weakening of intramolecular bond interactions (i.e., hydrogen bonds, aromatic, electrostatic and salt bridges), a decrease in the compactness of the hydrophobic core, an increase in the number of solvent-exposed hydrophobic side chains, longer and more hydrophilic loops and a reduction of metal binding affinity [3,8,9,10,20,23,24,25,26]. In some cold-active enzymes the quaternary structure is formed by a lower number of protomers (i.e., lower oligomerization state) compared to mesophilic and thermophilic homologues [27]. Nevertheless, in other cases, a higher oligomerization state was found to promote flexibility and activity at low temperatures [28,29,30,31]. Cold activity does not imply the coexistence of all these mechanisms, but one or some of them selected by evolution [3,8,9,10,20,23,24,25,26].

The comparison among cold-active enzymes and their mesophilic and thermophilic homologues highlights peculiar biochemical and biophysical features in the former [3,8,9,10,20,23,24,25,26]. Parameters useful to describe these features are summarized in Appendix A. Generally, cold-active enzymes are characterized by higher specific activity at low temperatures and lower optimal temperature of catalysis (*T_opt_*, 20–45 °C). However, some cold-active enzymes have a *T_opt_* similar to that of thermophilic homologues [32,33,34], indicating that the true hallmark of cold activity is rather the ability to maintain high activity at low temperature [12]. A distinctive element of cold-active enzymes is that enzymatic inactivation occurs at temperatures lower that the ones causing the loss of protein structure (*T_opt_* < unfolding transition midpoint temperature—*T_m_*−). This suggests that the active site is more thermolabile than the overall structure [3,8,9,10,20,23,24,25,26,35]. By contrast, in mesophilic and thermophilic enzymes inactivation coincides with the loss of structure (*T_opt_* = *T_m_*) [8,9]. Moreover, cold-active enzymes show low long-term thermostability compared to mesophilic and thermophilic counterparts. Indeed experiments performed with homologous α-amylases from psychrophilic, mesophilic and thermophilic organisms show that the psychrophilic one is completely inactivated after 60 min at 50 °C, while mesophilic and thermophilic enzymes maintain 60% and 90% of their activity after 100 min of incubation [8]. It is worth noting that the characterization of new enzymes from psychrophilic organisms has challenged these paradigms, as some enzymes combine activity in the cold with a stability comparable to that of their mesophilic and thermophilic counterparts [29,32,33,34,36,37,38]. A further side-effect of activity at low temperatures is the decrease in substrate specificity (higher *K_M_*) [3,8,9,10,20,23,24,25,26]. Moreover, the high flexibility and plasticity of the active site of cold-active enzymes can increase substrate promiscuity (i.e., the ability of one active site to catalyze different reactions) [39,40,41,42,43,44].

## 3. Sources of Cold-Active β-Galactosidases

Bioprospecting of marine Polar environments and deep-sea waters led to the identification of cold-active β-galactosidases from *Alteromonas*, *Alkalilactibacillus*, *Marinomonas* and *Pseudoalteromonas* species [29,45,46,47,48,49,50,51,52,53]. In addition, cold-active β-galactosidases were also isolated from psychrophilic and psychrotolerant microorganisms from different cold environments including Antarctic soil [54,55,56,57,58,59,60,61,62,63], glaciers [64] and deep lakes [65,66].

The physiological role of β-galactosidases in environments where lactose is missing or at very low concentrations [49] is still to be defined. Interestingly, the genome of some psychrophilic bacteria contains the genes encoding for two or more β-galactosidases belonging to different families. For instance, *Arthrobacter* sp. ON14 [59], *Arthrobacter* sp. B7 [54,61] and *Arthrobacter* sp. 32Cb [60,62] produce two β-galactosidases of the GH2 and GH42 families; four β-galactosidases, one GH35 and three GH42, are identified in the genome of *Cryobacterium* sp. LW097 [67]. In *Carnobacterium piscicola* BA and in *Alkalilactibacillus ikkense* the β-galactosidase encoding gene is in the same operon of that coding for α-galactosidase [49,68]. This evidence, together with the presence of other GHs and with the promiscuity of some β-galactosidases suggests that these enzymes might be involved in the degradation of polysaccharides containing β-galactosidic bonds (e.g., galactan, arabinogalactan) present in the environment, such as sugars from bacterial biofilms and marine algae [49,69].

## 4. Classification, Structure and Activity of Cold-Active β-Galactosidases

### 4.1. GH1 Family

Enzymes belonging to family GH1 display both β-galactosidase and β-glucosidase activities and employ a retaining mechanism of catalysis [70]. To date, only a few GH1s from psychrophilic bacteria have been characterized (Table 1). These enzymes show a *T_opt_* in the range from 25 °C to 40 °C and retain from ~10 to 57% of their highest activity at low temperatures [50,53,71,72]. In all cold-active GH1s, the β-glucosidase activity is the prominent one (Table 1), which makes them suitable for exploitation in the hydrolysis of cellulose and its derivatives. The cold-active GH1 with the highest β-galactosidase activity is the enzyme isolated from Baltic sea water by metagenomic analysis [50]. Interestingly, the mesophilic GH1 from the deep-sea bacterium *Bacillus* sp. D1. BglD1 shows high β-galactosidase activity and is active in lactose hydrolysis and in the production of galacto-oligosaccharides (GOS) [73], highlighting the importance of the amazing biodiversity of marine environments in enzyme discovery.

**Figure 3:**
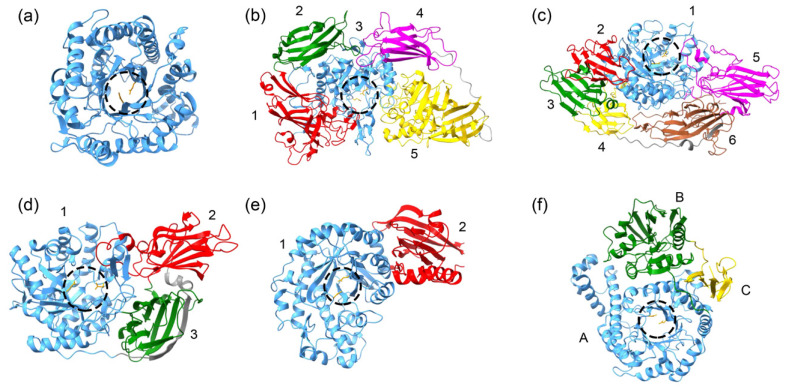
Tertiary structure of β-galactosidases representative of different glycosyl hydrolase families. The tertiary structure of β-galactosidases of family GH1 (**a**) (PDB: 5DT5, [31]), GH2 (**b**) (PDB: 1YQ2, [30]), GH35 group 1 (**c**) (PDB: 3OG2, [77]), GH35 group 2 (**d**) (PDB: 4E8C, [78]), GH35 group 3 (**e**) (PDB: 4D1I, [79]) and GH42 (**f**) (PDB: 1KWG, [80]) are shown. The catalytic domain is colored in blue; circles mark the region of the catalytic site and catalytic residues are highlighted in orange. Accessory domains are colored in different colors and named with numbers for GH2 and GH35 or letters for GH42 (for numbering and letters see text).

**Figure 4:**
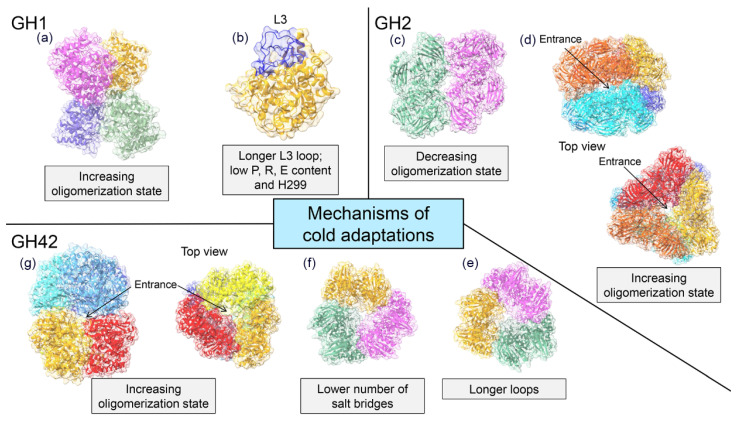
Cold adaptation mechanisms of β-galactosidases. Available 3D structures of cold-active β-galactosidases reveal several mechanisms of cold adaptation. In the GH1 family, high flexibility is due to a long-loop L3 and residue H299 in BglU (**a**) (PDB code: 3W53, [75]) and to the increase in the number of protomers in the quaternary assembly (increase of oligomerization state), as observed in EaBglA (**b**) (PDB: 5DT5, [31]). Changes in protein quaternary structure were observed also in the GH2 family. In ArthβDG (**c**) (PDB:6ETZ, [81]), the decrease of the multimerization state (from tetramer to dimer) enhances protein flexibility, whereas in C221-β-Gal (**d**) (PDB: 1YQ2, [30]) the increase of the oligomerization state (from tetramer to hexamer) promotes the formation of a large central cavity. Finally, in GH42 family, cold activity is accompanied by a decrease in the number of salt bridges, as observed in *Rahnella* sp. R3 (**e**) (PDB: 5E9A, [82]), longer loops, as observed in *H. lacusprofundi* (**f**) (PDB: 6LVW, [83]), and by an increase of the oligomerization state (from trimer to hexamer), as observed in M-βGal (**g**) (PDB:6Y2K, [29]) which supports the formation of a large central cavity. Each protomer is marked by a different color.

The tertiary structure of GH1 enzymes consists of a single (α/β)_8_ TIM–barrel containing two Glu catalytic residues that act as acid/base and nucleophile, respectively [31,75] (Figure 3a). Currently, the crystal structures of two cold-active GH1s from the Antarctic bacteria *Micrococcus antarcticus* (BglU) [75] and *Exiguobacterium antarcticum* B7 (EaBglA) [31] were solved. Although both enzymes share the (α/β)_8_ TIM–barrel fold and 44% sequence identity, they developed two different mechanisms of cold adaptation. The psychrophilic features of EaBglA are attributed to its peculiar tetrameric arrangement (Figure 4a), which increases the flexibility of the solvent-exposed regions [31]. On the other hand, sequence and structural analyses show that the low content of Pro, Arg, and Glu; the residue H299 within the tunnel connecting the enzyme surface to the catalytic site (Figure 4b); and the long-loop L3 are involved in the low temperature activity and thermolability of BglU [75,76].

### 4.2. GH2 Family

The largest number of known β-galactosidases, including the well characterized β-galactosidase from *Escherichia coli* (*Ec-*βgal) encoded by *lacZ* [84], belongs to the GH2 family. Cold-active GH2 β-galactosidases hydrolyze β (1-4) glycosidic bond in β-d-galactosides and are characterized by a *T_opt_* in the range 10 °C–50 °C and by low long-term thermostability (Table 2). The activity of some cold-active GH2s is positively modulated by Mn^2+^, Mg^2+^, Na^+^ and K^+^ [46,54,55,56,57,59,60,85], whereas GH2s from *Arthrobacter* sp. SB, *Arthrobacter* sp. 32 cB, *Paracoccus* sp. 32d and *Pseudoalteromonas* sp. 22b are inhibited by glucose or galactose, the product of lactose hydrolysis [57,60,63,85].

From a structural point of view, all GH2s are organized in a five-domain tertiary structure (Figure 3b): domain 1 is a sugar-binding domain with jelly-roll fold; domains 2 and 4 are immunoglobulin-like-β-sandwich domains; domain 3, which contains two Glu catalytic residues, has the (α/β)_8_ TIM–barrel fold; domain 5, named “β-galactosidase small chain”, has a typical (α/β) fold [30,81,84,86]. The well-known *Ec-*βgal has a homo-tetrameric quaternary structure [84]. Interestingly, the available 3D structures of all cold-active GH2s reveal quaternary arrangements different from that of *Ec-*βgal.

Cold-active GH2s from *Paracoccus* sp. 32d (Par_DG) and *Arthrobacter* sp. 32cB (ArthβDG) are dimers in their native form (Figure 4c). This arrangement defines a large solvent-exposed area and increases the flexibility of the protein surface compared to the tetrameric *Ec-*βgal [81,86]. Par_DG and ArthβDG evolved the same solution for cold adaptation (decrease of the oligomerization state) but employed two different molecular mechanisms. Indeed, in ArthβDG dimers are stabilized by hydrogen bonds and hydrophobic interactions between amino acidic residues of domains 1 and 5 of adjacent subunits [81], whereas in Par_DG dimers formation rely on the properties of domain 5 that is more compact and smaller than that of the mesophilic enzyme [86]. By contrast, the cold-active GH2 β-galactosidases from *Arthrobacter* sp. C2-2 (C221-β-Gal) adopts a compact hexameric quaternary structure (Figure 4d), which enhances the number of channels and cavities and allows for the formation of an internal catalytic cavity, accessible through three channels [30].

### 4.3. GH35 Family

GH35 β-galactosidases are multi-domain enzymes, which can hydrolyze β (1-4), (1-3) and (1-6) glycosidic bonds in β-d-galactosides such as disaccharides (e.g., lactose), oligosaccharides, glycoproteins and glycolipids [87,88,89]. Currently, only three cold-active GH35s have been characterized from the psychrophilic bacteria *Carnobacterium piscicola* BA, *Cryobacterium* sp. LW097 and *Arthrobacter* sp. B7 [90,91]. The enzyme from *C. piscicola* BA has a *T_opt_* of 40 °C, retains 20% of its highest activity at 5 °C and it is endowed with low long-term thermostability [91]. The GH35 from *Arthrobacter* sp. B7 is a dimer and it is specific towards β (1-4) and β (1-3) glycosidic bonds. Unfortunately, no information regarding *T_opt_* and activity at low temperatures is available [90]. The best characterized cold-active enzyme of this family is the β-galactosidase from *Cryobacterium* sp. LW097 (Bgal436), which shows a *T_opt_* of 40 °C and retains 40% of its maximum activity at 5 °C [67]. Although, Bgal436 hydrolyzes both *o*-NP-β-d-galactopyranoside and lactose with a *K_M_* of 2.1 and 13.1 mM at 5 °C, respectively, its preferred substrate is allolactose (β (1-6) glycosidic bonds) [67].

Based on available 3D structures, GH35s can be divided in three different groups (Figure 3), all with the same (α/β)_8_ TIM–barrel fold of the catalytic domain. Enzymes in group 1 are monomers made-up of a central catalytic domain surrounded by a horseshoe of five anti-parallel β-sandwich domains (Figure 3c) [77,92,93]. By contrast, enzymes in group 2 are dimers composed by three different domains, where domain 1 is the catalytic one, and domains 2 and 3 are all-β-domains (Figure 3d) [78,94,95]. Finally, the GH35 from *Cellvibrio japonicus* (group 3), folds in a two-domain architecture in which the catalytic domain is followed by a small C-terminal domain with a mixed α/β structure (Figure 3e) [79]. Although the 3D structures of cold-active GH35s are not available, sequence analysis indicate that these enzymes belong to group 2. Indeed, they share high sequence identity (>37%) with the enzymes in group 2 and low sequence identity (<25%) with those belonging to group 1 and 3.

### 4.4. GH42 Family

Most GH42 enzymes are isolated from extremophilic microorganisms [48,61,62,64,65,66,80,91,96,97,98,99]. All cold-adapted GH42s are characterized by heterogeneous long-term thermal stability and *T_opt_* (from 20 °C to 60 °C —Table 3—). Interestingly, cold-active GH42s from Arctic and Antarctic *Marinomonas* sp. display an unusual thermal stability similar to those of mesophilic counterparts (Table 3) [48]. All enzymes hydrolyze the β (1-4) glycosidic bond in β-d-galactosides (Table 3). The cold-active GH42s from *Carnobacterium maltaromaticum* and from *Planococcus* sp. SOS Orange are active also on β-d-fucosides [91,96], whereas the enzyme identified by metagenomic analysis of topsoil of Daqing oil field, in the north of China, have the broadest substrate specificity (Table 3) [66]. Currently, only the cold-active GH42 from *Marinomonas sp.* BSi20414 is described to be selective for the β (1-3) glycosidic bond in β-d-galactosides [48]. Cold-active GH42s from *Cryobacterium* sp. LW097 are able to hydrolyze lactose, galactobiose, lactulose and allolactose (β (1-6) galactosidic bond) [67].

GH42 β-galactosidases are three-domain enzymes (domain A, B and C —Figure 3d—). Domain A is the catalytic one, contains the two Glu catalytic residues and is organized in a (α/β)_8_ TIM–barrel fold, domain B is the so-called trimerization domain, involved in the stabilization of the quaternary structure and domain C consists of an anti-parallel β sandwich with unknown function [80,82,98,99,100]. Most GH42 enzymes have a trimeric quaternary structure (Figure 4e,f) except for the cold-active GH42 from *Marinomonas* sp. ef1 (M-βGal) which has a hexameric arrangement (dimer of trimmers —Figure 4g—) [29]. Structural analysis reveals three different mechanisms of cold adaptation in the enzymes of this family [29,82,83]. Activity at low temperature of the trimeric *Rahnella* sp. R3 enzyme might rely on a lower number of salt bridges and on higher flexibility than the mesophilic and thermophilic homologues [82]. The GH42 from *H. lacusprofundi* is a trimer that couples adaptation to high salt as well as to cold environments. Indeed, it presents an acidic surface, typical of halophilic enzymes, and two long and flexible loops localized in domain B and C (Figure 4f) [83]. The hexameric arrangement of M-βGal, together with the absence of a zinc binding site, create an internal catalytic cavity accessible from five gates (two at the apices and three at the equatorial region of the hexamer) [29]. The internal catalytic cavity might modulate the substrate accessibility and affinity with a mechanism similar to that proposed for C221-β-Gal, highlighting a new strategy of cold adaptation. The peculiar quaternary structure might account for the cold activity and the robustness of M-βGal.

## 5. Industrial Applications of Cold-Active β-Galactosidases

The hydrolytic and transglycosylation activities of β-galactosidases make these enzymes promising from an industrial point of view [14,101,102]. The main advantages of using cold-active β-galactosidases are envisaged in the preservation of heat-labile compounds [4,12]. In this review, we describe the state-of art in the use cold-active β-galactosidases in lactose hydrolysis and in the production of GOS.

### 5.1. Hydrolysis of Lactose in Milk

The hydrolysis of lactose in milk can be carried out by either chemical or enzymatic treatment. β-galactosidases are widely used in the production of lactose-free dairy products, because by-products are avoided and the chemical-physical characteristics of milk are not altered [14,101,102,103]. These products are dedicated to lactose-intolerant people, who have a deficiency of β-galactosidases in their digestive system [104]. Moreover, β-galactosidases are used in ice cream and in condensed milk production to avoid lactose crystallization and to enhance the sweetness and creaminess of these products [101,102]. Lactose-free milk is produced adding soluble β-galactosidases either during milk storage (batch process) or after UHT treatment (aseptic process) [103]. Generally, these processes are carried out by using mesophilic enzymes such as the *Kluyveromyces lactis* and *Aspergillus oryzae* β-galactosidases, which are active at refrigeration temperature [105]. The biochemical features of cold-active enzymes, in particular their high activity at low temperatures and thermolability, make them suitable for the batch process, carried out under slow shaking for 24 h, at 4–8 °C, before pasteurization and packaging [103]. Several cold-active β-galactosidases have been tested for their performances in the hydrolysis of milk lactose (Table 4). Unfortunately, the lack of standardization makes it difficult to compare results obtained in different laboratories. GH2 *Pseudoalteromonas* sp. 22b and *Pseudoalteromonas* sp. 79b β-galactosidases were immobilized on chitosan and sepharose, respectively, to improve their activity and stability during lactose hydrolysis reactions. Immobilization was found to increase the stability of both enzymes, as well as the catalytic performances of the *Pseudoalteromonas* sp. 22b enzyme [85,106].

### 5.2. Hydrolysis of Lactose in Cheese Whey

Cheese whey (CW) is one of the main by-products of the cheese-making processes [109]. CW is the liquid phase obtained after casein coagulation and curd separation and it contains lactose, proteins, lipids and mineral salt [109]. CW proteins are valuable and find several applications in food and feed industries [110]. By contrast, lactose is the most polluting component because of its high value of biochemical oxygen demand. Since its release in the environment is forbidden, CW is used by the food and feed sectors and as a source of lactose for the production of high value compounds by microbial fermentation or by chemical modifications [109,111,112]. In this context, β-galactosidases are used in the pre-treatment of CW to obtain glucose and galactose, which are more suited than lactose as substrates for microbial growth [109,111,112]. The hydrolysis of CW lactose in the presence of mesophilic and thermophilic β-galactosidases (i.e., *Kluyveromyces fragilis*, *Aspergillus oryzae* and *Sulfolobus solfataricus*) is carried out at 35–55 °C [113,114,115]. The marine cold-active GH2 from *Pseudoalteromonas haloplanktis* was employed for the hydrolysis of lactose in CW permeate (i.e., CW without proteins) to produce d-tagatose, which is a natural low-calorie sweetener [116]. In this process, lactose hydrolysis was performed at 23 °C, a temperature that does not require cooling or heating of the tank.

Unfortunately, this is to date the only report about the use of a cold-active enzyme. Nevertheless, the information derived from studies about the hydrolysis of milk lactose (e.g., reaction conditions, temperature of hydrolysis etc.) can be applied to design a sustainable process for lactose hydrolysis in CW permeate.

### 5.3. Synthesis of Oligosaccharides

GOS are prebiotics that stimulate the growth of beneficial gut bacteria (e.g., *Lactobacilli* or *Bifidobacteria*) and prevent the colonization of pathogenic bacteria in the gastrointestinal tract. Moreover, GOS are used in the cosmetic and in food industries as additives and sweeteners, respectively [14,101,102]. The use and potential of β-galactosidases for the synthesis of GOS and glycan conjugates has been recently reviewed by Lu and coauthors [14]. Although high temperatures increase lactose solubility in GOS production [117,118,119,120], a few papers reported the use of cold-active β-galactosidases in the synthesis of GOS [45,60,121], notably the enzymes from the marine bacteria *Alteromonas* sp. ANT48 and *Marinomonas sp.* BSi20414 (MaBGA) [45,121]. Despite GOS usually containing β-1,4 and β-1,6 linkages [14,101,102], the transglycosylation reaction carried out in the presence of MaBGA produces a trisaccharide with uncommon β-1,3 linkages [121].

The cold-active GH2 from the marine *Pseudoalteromonas* sp. 22b is active in the glycosylation of short chain alcohols (C3–C6) to yield alkyl glycosides, which can be used in the cosmetic industry and/or as building block in the synthesis of pharmaceutical products. These reactions were carried out at 30 °C for 24 h [122]. For comparison, one could consider that the synthesis of butylgalactoside by *A. oryzae* β-galactosidase was performed at 50 °C [123].

Moreover, the cold-active GH2 from *Arthrobacter* sp. 32bc synthesizes, by transglycosylation at 30 °C, hetero-oligosaccharides such as lactulose, galactosyl-xylose and galactosyl-arabinose [60]. Lactulose (4-O-β-d-galactopyranosyl-β-d-fructofuranose) is used in food as a prebiotic and in medicine in the treatment of hepatic encephalopathy and constipation. The enzymatic synthesis of lactulose is more advantageous than the chemical one, because the latter requires harsh conditions (alkaline pH —10.5–11 and high temperatures —70–100 °C), which degrade lactose producing different by-products [60]. Among mesophilic enzymes, the best yield of lactulose is obtained by the use of the *A. oryzae* β-galactosidase at 40 °C [124].

## 6. Conclusions

*Cold adaption mechanisms*. The number of enzymes identified from psychrophilic and psychrotollerant organisms is constantly growing. The biochemical data available on β-galactosidases led us to reconsider the hallmarks of cold activity. Several authors proposed low *T*_opt_ values as the main distinctive feature of cold adaptation. However, the comparison of cold-active β-galactosidases suggested that this parameter is the most heterogeneous, spanning from 10 °C to 60 °C. Long-term thermal stability, usually lower compared to that of mesophilic counterparts, is very variable as well (Table 1, Table 2 and Table 3). One cannot completely exclude that this heterogeneity might be due to an incomplete evolutionary adaptation to the cold [10]. On the other hand, the ability of these enzymes to maintain their activity in the cold is clearly the true label of cold activity. Based on this evidence also the β-galactosidases from the mesophilic *Kluyveromyces lactis* and *Aspergillus oryzae* [105] and from the thermophilic *Pyrococcus furiosus*, which retains 8% of its maximum activity at 0 °C [125], are to be considered cold-active enzymes. To shed light on cold adaptation mechanisms, a useful, still yet unexplored, parameter is the *T_m_* value. Indeed, the comparison between the *T*_opt_ and the *T_m_* values gives information on the thermolability of the active site and the catalytic intermediates [3,8,9,10,20,23,24,25,26,35]. Unfortunately, due to the paucity of *T_m_* data, such kind of comparison is not possible for cold-active β-galactosidases, suggesting that a more systematic approach is required in the study of cold active enzymes.

Even more complex is the search for structural elements responsible of cold adaptation. Despite the paucity of available 3D structure of cold-active β-galactosidases, at least four different mechanisms of cold adaptation can be observed (Figure 4). Besides the canonical adaptive structural changes (longer loops, less salt bridges etc.) some of these mechanisms result in modifications of the protein topology and of the quaternary structure. In detail, Par_DG and ArthβDG lowered the oligomerization state increasing the solvent exposed surface and thereby flexibility [81,86]. Nevertheless, also higher multimerization could be considered a strategy of cold adaptation, as it may increase the flexibility of the solvent-exposed region as described for EaBglA [31] or create a large catalytic cavity, which modulates the substrate accessibility as described for C221-β-Gal and M- βGal [29,30].

It is interesting to note that this heterogeneity is also observed among members of the same protein family, suggesting that two enzymes, phylogenetically distant, could give rise to different strategies if exposed to similar selective pressure. Overall, the structural reasons of cold adaptation seem to be inspired by thermodynamic requirements to enhance the catalytic efficiency at low temperatures rather than to derive from a common signature.

*Biotechnological exploitation of cold-active β-galactosidases*. The importance and the advantage of cold-active enzymes in industrial application are known and their potentiality is reported in several reviews [3,12,13]. Cold-active β-galactosidases from psychrophilic and mesophilic organisms might play a key role in food industries in the production of lactose free products and prebiotics. However, the lack of standardization makes it difficult to compare the hydrolytic activity of different enzymes. Therefore, a set of parameters, including milk preparation, enzyme concentration and temperature of hydrolysis, have to be defined to compare different biocatalytic processes and to improve their industrial exploitation.

The transglycosylation activity of cold-active β-galactosidases can be applied in the synthesis of GOS, heterooligosaccharides and alkyl glycosides. Since transglycosylation takes place with an enzyme dependent mechanism, the discovery of new cold-active β-galactosidases could drive the development of new products and the design of new processes that aim to replace chemical treatments with enzymatic ones.

In conclusion, cold-active β-galactosidases are still poorly explored. However, the structural and functional heterogeneity they display can be useful to shed light on the molecular bases of cold adaptation and for their biotechnological exploitation. In this context, the marked biodiversity of marine environments could play a key role in the discovery of new cold-active β-galactosidases with industrial and scientific interest.

## Figures and Tables

**Table 1:** Biochemical features of family GH1 cold-active β-galactosidases. Enzymes from marine microorganisms are in bold.

Source	*T_opt_* (°C)	Cold Activity ^1^ (%)	Residual Activity (%)	Substrate Specificity ^2^	References
***Alteromonas* sp. L82**	40	9.4 (4 °C)	~10 (3 h at 40 °C)	Cellobiose (100%) Lactobiose (3.6%) *p*-NP-β-d-glucopyranoside (100%) *o*-NP-β-d-glucopyranoside (120.7%) *p*-NP-β-d-cellobioside (8%) *p*-NP-β-d-galactopyranoside (9.2%) *o*-NP-β-d-galactopyranoside (13%) *p*-NP-β-d-xylopyranoside (0.9%)	[53]
**Baltic sea sediment**	40–45	10 (5 °C)	0 (30 min at 40 °C)	*p*-NP-β-d-glucopyranoside (130%) *p*-NP-β-d-fucopyranoside (133%) *p*-NP-β-d-galactopyranoside (100%) *o*-NP-β-d-galactopyranoside (82%) *p*-NP-β-d-cellobioside (59%) *p*-NP-β-d-xylopyranoside (4%)	[50]
*Exiguobacterium antarcticum B7*	30	25 (5 °C)	~20 (72 h at 30 °C)	*p*-NP-β-d-glucopyranoside (100%) *p*-NP-β-d-cellobioside (50.9%) *p*-NP-β-d-galactopyranoside (2.3%) *p*-NP-α-d-glucopyranoside (1.2%) *p*-NP-β-d-mannopyranoside (0.5%)	[71]
*Micrococcus antarcticus*	25	27 (0 °C)	~20 (60 min at 35 °C)	*p*-NP-β-d-glucopyranoside (100%) *p*-NP-β-d-galactopyranoside (32.2%)	[72]
***Marinomonas*** **sp. MWYL1**	40	20 (5 °C)	~75 (60 min at 40 °C)	*p*-NP-β-d-glucopyranoside (100%) *p*-NP-β-d-galactopyranoside (26.5%)	[74]

^1^. Relative activity obtained at the temperature indicated in brackets was calculated as the percentage of the activity at *T_opt_.*^2^. Relative activity reported in the reference is shown in brackets.

**Table 2:** Biochemical features of cold-active β-galactosidase of family GH2. Enzymes from marine microorganisms are in bold.

Source	*T_opt_* (°C)	Cold Activity ^1^ (%)	Residual Activity (%)	Substrate Specificity ^2^	References
***Alkalilactibacillus ikkense***	30	~60 (0 °C)	~ 20 (5 h at 30 °C)	*p*-NP-β-d-galactopyranoside (100%) *p*-NP-β-d- fucopyranoside (4%)	[49]
***Alteromonas* sp. ANT48**	50	~30 (0 °C)	~20 (3 h at 60 °C)	*o*-NP-β-d-galactopyranoside (100%) *p*-NP-β-d-galactopyranoside (14%)	[45]
***Alteromonas*** **sp.** **ML117**	30–35	~20 (5 °C)	0 (1 h at 30 °C)	*o*-NP-β-d-galactopyranoside (100%) *p*-NP-β-d-galactopyranoside (31%)	[51]
***Alteromonas*** **sp. ML52**	35	~20 (5 °C)	~10 (1 h at 35 °C)	*o*-NP-β-d-galactopyranoside (100%) *p*-NP-β-d-galactopyranoside (12.8%)	[52]
*Arthrobacter psychrolactophilus* F2	10	~90 (0 °C)	~20 (2 h at 35 °C)	*o*-NP-β-d-galactopyranoside	[58]
*Arthrobacter* sp. ON14	15	~30 (0 °C)	~40 (2 h at 40 °C)	*o*-NP-β-d-galactopyranoside	[59]
*Arthrobacter* sp. 20B	25	~30 (0 °C)	~30 (1 h at 45 °C)	*p*-NP-β-d-galactopyranoside	[56]
*Arthrobacter* sp. 32cB	28	~30 (5 °C)	~10 (8 h at 35 °C)	*p*-NP-β-d-galactopyranoside (100%) *p*-NP-β-d-fucopyranoside (4%)	[60]
*Arthrobacter* sp. B7	40	~25 (10 °C)	~50 (2 h at 40 °C)	*p*-NP-β-d-galactopyranoside (100%) *p*-NP-β-d-galuronide (4%)	[54]
*Arthrobacter* sp. C2–2	40	~15 (5 °C)	~0 (1 h at 45 °C)	*o*-NP-β-d-galactopyranoside	[55]
*Arthrobacter* sp. SB	18	~50 (0 °C)	~50 (2 h at 40 °C)	*o*-NP-β-d-galactopyranoside	[57]
*Flavobacterium* sp. 4214	42	~10 (15 °C)	~35 (2 h at 40 °C)	*o*-NP-β-d-galactopyranoside (100%) *p*-NP-β-d-fucopyranoside (39%)	[63]
***Pseudoalteromonas haloplanktis* TAE 79**	45	~18 (7 °C)	0 (1 h at 45 °C)	*o*-NP-β-d-galactopyranoside	[46]
***Pseudoalteromonas* sp. 22b**	40	~10 (0 °C)	~90 (1 h at 40 °C)	*o*-NP-β-d-galactopyranoside (100%) *p*-NP-β-d-galuctoronide (1.5%)	[47]

^1^. Relative activity obtained at the temperature indicated in brackets was calculated as the percentage of the activity at *T_opt_.*^2^. Relative activity reported in the reference is shown in brackets.

**Table 3:** Biochemical features of cold-active β-galactosidase of family GH42. Enzymes from marine microorganisms are in bold.

Source	*T_opt_* (°C)	Cold Activity ^1^ (%)	Residual Activity (%)	Substrate Specificity ^2^	References
*Arthrobacter* sp. 32cB	50	~18 (0 °C)	N.A.	*p*-NP-β-d-galactopyranoside (100%) *p*-NP-β-d-glucopyranoside (1.4%)	[62]
*Arthrobacter* sp. B7	50	~50 (4 °C)	~0 (15 min at 50 °C)	*o*-NP-β-d-galactopyranoside Lactose	[61]
*Carnobacterium maltaromaticum*	30	~10 (0 °C)	~10 (30 min at 35°C)	*o*-NP-β-d-galactopyranoside (100%) *p*-NP-β-d- fucopyranoside (10.1%)	[91]
*Cryobacterium* sp. LW097 (Bgal322)	25	~60 (5 °C)	~32 (12 h at 35 °C)	*o*-NP-β-d-galactopyranoside (100%) *p*-NP-β-d-galactopyranoside (69%)Lactose (5%) Galactobiose (100%) Lactulose (11%) Allolactose (44%)	[67]
*Cryobacterium* sp. LW097 (Bgal435)	30	~60 (5 °C)	~13 (12 h at 35 °C)	*o*-NP-β-d-galactopyranoside (100%) *p*-NP-β-d-glucopyranoside (34%)Lactose (34%) Galactobiose (6%) Lactulose (8%) Allolactose (100%)	[67]
*Cryobacterium* sp. LW097 (Bgal2567)	35	~40 (5 °C)	~14 (12 h at 35 °C)	*o*-NP-β-d-galactopyranoside (100%) *p*-NP-β-d-galactopyranoside (126%) Lactose 44%) Galactobiose (90%) Lactulose (97%) Allolactose (100%)	[67]
*Halorubrum lacusprofundi*	50	~10 (<10 °C)	N.A.	*o*-NP-β-d-galactopyranoside	[65]
***Marinomonas*** **sp. BSi20414**	60	~10 (10 °C)	76 (6 h at 40 °C)	*p*-NP-β-d-galactopyranoside	[48]
***Marinomonas*** **sp. ef1**	55	23 (5 °C)	25 (4 days at 50 °C)	*o*-NP-β-d-galactopyranoside	[29]
*Planococcus* sp. L4	20	~30 (0 °C)	20 (1 h at 40 °C)	*o*-NP-β-d-galactopyranoside	[97]
*Planococcus* sp. SOS orange	40	~10 (0 °C)	30 (2 h at 40 °C)	*o*-NP-β-d-galactopyranoside (100%) *p*-NP-β-d-fucopyranoside (6.1%)	[96]
*Rahnella* sp. R3	35	27 (4 °C)	~90 (2 h at 35 °C)	*o*-NP-β-d-galactopyranoside (100%) Lactose	[64]
Topsoil of Daqing oil field	40	~10 (0 °C)	~17 (1 h at 40 °C)	*o*-NP-β-d-galactopyranoside (100%) *p*-NP-β-d-glucoronide (49.5%) *p*-NP-β-d-arabinoside (52.8%) *p*-NP-β-d-mannoside (61.3%)	[66]

^1^. Relative activity obtained at the temperature indicated in brackets was calculated as the percentage of the activity at *T_opt_.*^2^. Relative activity reported in the reference is shown in brackets.

**Table 4:** Cold active β-galactosidases in the hydrolysis of milk. Enzymes from marine microorganisms are in bold.

Source	T (°C)	Time (h)	Amount of Enzyme	Hydrolysis Yield (%)	References
***Alteromonas*** **sp. ML52**	4	24	44.5 U	90	[52]
*Arthrobacter* *psychrolactophilus* F2	10	24	10 U/mL	80	[58]
*Arthrobacter* sp. *ON14*	4	8	5.08 U/mL	100	[59]
*Arthrobacter* sp. 32cB	10	24	2 U/mL	90	[60]
*Arthrobacter* sp. SB	2.5	7.5	N.A.	80	[57]
*Aspergillus oryzae (EYL)*	2	24	0.1% (*w*/*v*)	85.23	[105]
*Cryobacterium* sp. LW097 (Bgal322)	4	48	5 U/mL	8.4	[67]
*Cryobacterium* sp. LW097 (Bgal435)	4	48	5 U/mL	5.1	[67]
*Cryobacterium* sp. LW097 (Bgal436)	4	48	5 U/mL	N.A.	[67]
*Cryobacterium* sp. LW097 (Bgal2567)	4	48	5 U/mL	7.8	[67]
***Halomonas sp. S62***	7	24	0.15 U/10 µL	60	[107]
*Kluyveromyces fragilis (LYL)*	2	24	0.1% (*w*/*v*)	82.96	[105]
*Kluyveromyces lactis (DYL)*	2	24	0.1% (*w*/*v*)	99.08	[105]
*Kluyveromyces lactis (VYL)*	2	24	0.8% (*w*/*v*)	98.59	[105]
*Paracoccus* sp. 32d	10	11	1 U/mL	91	[63]
*Planococcus sp. L4*	5	1	2.5 ug	36	[97]
***Pseudoalteromonas*** ***haloplanktis* TAE 79**	4	50 min	1.3 U	33	[46]
*Pseudoalteromonas* sp. 79b	10	8	1U	40	[106]
*Pseudoalteromonas* sp. 22b	4	48	30 U	65	[85]
*Topsoil of Daqing oil field*	4	1	1 U	4.2	[66]
*Lactococcus lactis*	4	9	0.14 U	98	[108]
Immobilized					
***Pseudoalteromonas*** **sp. 22b**	4	24	30 U per g of lactose	93	[85]
***Pseudoalteromonas* sp. 79b**	10	8	1U	40	[106]

**Figure A1:**
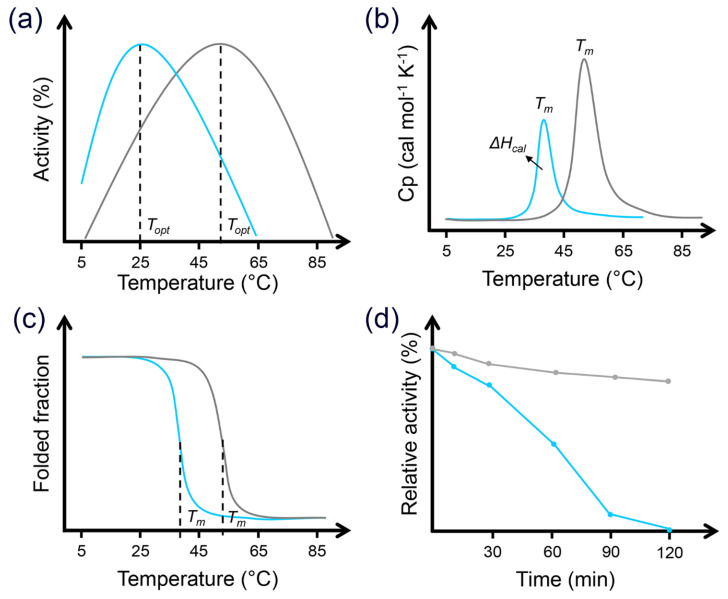
Biochemical and biophysical features of a generic cold-active enzyme and of a mesophilic homologue. (**a**) The optimum temperature of catalysis of psychrophilic (cyan) and mesophilic enzymes (grey). (**b**,**c**) Thermal unfolding of cold-active enzymes. (**b**) Thermograms obtained by DSC. Cold-active enzymes are characterized by lower *Tm* (top of the peak) and Δ*H*cal (area under the peak). (**c**) Thermal unfolding profile obtained by circular dichroism or fluorescence spectroscopies. (**d**) Long-term thermal stability obtained incubating the enzymes at temperatures close to the *Topt.*

## Data Availability

No new data were created or analyzed in this study. Data sharing is not applicable to this article.

## Appendix A

The temperature dependence of the activity of enzymes is a key property in the study of cold adaptation. The optimum temperature of catalysis (*T_opt_*) is determined by measuring enzyme activity at fixed temperature and conditions. Thus, the enzyme activity or relative activity is plotted as a function of temperature to obtain the graph shown in Figure A1a. Generally, the comparison of the optimum profiles of cold-active enzymes with those of mesophilic homologues pointed out that cold-active enzymes are more active at low and moderate temperatures than their mesophilic counterparts (Figure A1a). Moreover, usually the highest activity of cold-active enzymes is displayed at 20–45 °C, whereas that of mesophilic enzymes is displayed at 50–60 °C (Figure A1a) [9,10,44].

Thermal unfolding can be determined through differential scanning calorimetry (DSC) or through circular dichroism and/or fluorescence spectroscopies. DSC measures the heat absorbed in the medium during unfolding (Figure A1b) [9]. This technique allows us to determine (i) the *T_m_*, corresponding to the temperature of the top of the transition; (ii) the calorimetric enthalpy (*ΔH_cal_*) obtained calculating the area of the peak and related to enthalpic contribution; (iii) the cooperativity of the unfolding transition [9,126]. Similarly, *T_m_* can be determined by monitoring changes in secondary or tertiary structure, at fixed wavelength, in a given temperature range using CD (Far-UV and Near-UV) or intrinsic fluorescence spectroscopies [127,128]. With these techniques it is possible to calculate the *T_m_*, which corresponds to the flex of the sigmoidal curve and the cooperativity of the unfolding transition (Figure A1c) [127,128].

Another useful parameter is the long-term thermostability measured by incubating the enzyme at fixed temperature and monitoring the decrease of activity and/or structure over the time (Figure A1d) [9]. Cold-active enzymes are characterized by low long-term thermostability and maintain their activity for a few hours when incubated at temperatures near the *T_opt_*. By contrast, mesophilic and thermophilic enzymes are stable for a long time (some hours or days) at the same temperature [9].
